# Fangjing decoction relieves febrile seizures-induced hippocampal neuron apoptosis in rats via regulating the Akt/mTOR pathway

**DOI:** 10.1042/BSR20181206

**Published:** 2018-10-31

**Authors:** Xian-ke Xu, Sun-yao Wang, Ying Chen, Lu Zhan, Zheng-yang Shao, Long Lin, Wei-chao Yan, Shu-fen Mei

**Affiliations:** Department of Pediatrics, Hangzhou Red Cross Hospital, Hangzhou 310002, China

**Keywords:** AKT/mTOR pathway, brain injury, febrile seizures, Fangjing decoction, GABA

## Abstract

**Background**: Fangjing decoction is a Traditional Chinese Medicine that exhibits anticonvulsive effects in treating febrile seizures (FS). Its action mechanism and the regulation on Akt/mammalian target of rapamycin (mTOR) pathway were revealed in the present study.

**Methods**: FS model was established in Sprague–Dawley rats with or without Fangjing decoction treatment. On day 5, following initiation of drug treatment, seizures were monitored. Hippocampal neuron apoptosis was assessed using terminal dUTP nick end-labeling method. The levels of Bax, protein kinase B (Akt), phospho-Akt (p-Akt), mTOR, and p-mTOR proteins were analyzed using Western blotting. The content of hippocampal γ-aminobutyric acid (GABA) was measured by using ELISA assay.

**Results**: Compared with the control group (*n*=8), Fangjing decoction effectively shortened escape latency and duration of FS and decreased the frequency of FS in rats (*n*=8). Concomitantly, the apoptosis of hippocampal neurons, as well as Bax protein levels were also decreased in FS rats which were treated with Fangjing decoction. In addition, the Akt/mTOR signaling was found to be activated in rat hippocampus following FS, as evidenced by increased p-Akt and p-mTOR, while Fangjing decoction could inhibit the activation of Akt/mTOR signaling. Furthermore, the low GABA content in rat hippocampus following FS was significantly elevated by Fangjing decoction treatment. More importantly, SC79, a specific activator for Akt, apparently attenuated the protective effects of Fangjing decoction on FS rats.

**Conclusion**: These results suggest that Fangjing decoction protects the hippocampal neurons from apoptosis by inactivating Akt/mTOR pathway, which may contribute to mitigating FS-induced brain injury.

## Introduction

Febrile seizures (FS) are the most common type of convulsive events affecting up to 2–5% in infants as well as young children. One-third of patients with FS, especially prolonged seizures, experience recurrent FS [1,2]. It has been reported that 2–8% of the infants and young children experiencing recurrent FS suffer temporal lobe epilepsy that was associated with memory deficits [3]. Consistent studies exist with regard to the increased risk for developing brain injury by prolonged or recurrent FS, leading to hippocampus and cortex neuron apoptosis, which in turn can result in cognitive dysfunctions.

It is generally known that protein kinase B (Akt) plays a critical role in the manipulation of diverse cellular functions, such as metabolism, proliferation, survival, transcription, and protein synthesis [4]. The mammalian target of rapamycin (mTOR) is another serine/threonine kinase that controls protein synthesis related to cell growth and proliferation [5]. The Akt/mTOR signaling pathway has been reported to play a significant role in the regulation of various neuronal functions, such as synaptic plasticity and neurogenesis [6]. Recently, accumulating evidence has strongly implied that inhibition of the kinase activity of Akt might promote FS-mediated hippocampus neuronal apoptosis, suggesting that Akt/mTOR pathway is involved in FS-induced neuron injury [7]. Furthermore, γ-aminobutyric acid (GABA) is the chief inhibitory neurotransmitter that has been detected in the peripheral and central nervous systems, where it causes severe neurological and neuromuscular symptoms [8]. There are increasing evidences emerging to suggest that low expression level of GABA is one of the important causes of seizures [9]. For instance, Mackenzie et al. [10] reported that chronic stress-induced GABA down-regulation in the hippocampus of mice might contribute to stress-induced seizure susceptibility. Therefore, in the present study, we focus on the Akt/mTOR pathway and GABA concentration as the indicators in assessing the curative effect of FS.

In clinical, many anticonvulsant drugs have been discovered, including phenobarbital, valproic acid, carbamazepine, and phenytoin; whereas, due to the severe adverse/toxicity effects or poor efficacy of these agents, safer and more efficacious drugs are urgently needed for prevention and treatment of FS [11,12]. Traditional Chinese Medicine has long been used to treat FS. In a previous clinical study, we found that Fangjing decoction which is a combination of multiple classical Chinese prescriptions could prolong the latency of recurrence of febrile convulsions, shorten duration, and lessen the degree of convulsions [13]. Fangjing decoction is a combination of nine herbs generally used in Traditional Chinese Medicine by decocting them altogether, and the specific compositions and their proportion, main functions and bioactive component have been listed in Table 1. The precise anticonvulsive mechanism of Fangjing decoction; however, is still unknown.

**Table 1:** The compositions of Fangjing decoction

Compositions	Weight (g)	Main functions	Bioactive component	References
Radix Pseudostellariae	10	Increases immunologic function, fatigue resistance, and hypoxia tolerance	Ginseng saponin	[14]
Atractylodes Rhizome stir-fried with wheat bran	10	Anti-inflammation and antitumor gastrointestinal tract regulation	Atractylenolide-I	[15]
Poria Cocos	10	Antiepileptic activity	Total triterpenes	[16]
Radix Paeoniae alba	10	Anticonvulsive effect	Paeoniflorin	[17]
Gastrodia Elata	10	Antiepileptic effect	Gastrodin	[18]
Uncaria Rhynchophylla	10	Antiepileptic effect	Rhynchophylline	[19]
Acori graminei Rhizoma	10	Neuroprotective effects	Asarone	[20]
Concha Haliotidis	10	Antioxidant effect and anti-inflammation	Abalone shell	[21]
Liquorice	5	Protection of the nervous system	Triterpenoid saponin	[22]

In the present study, the rat model of FS was used to further evaluate the underlying antiseizures roles of Fangjing decoction. Additionally, we investigated whether Fangjing decoction modulated the Akt/mTOR signaling pathway to determine the potential mechanism of its antiseizures effects.

## Methods

### Rat model of FS

Male SPF grade Sprague–Dawley (SD) rats aged 10 days, obtained from the Zhejiang Chinese Medical University and fed with standard rodent chow under specific pathogen-free conditions, were used in our study. By using heated water-bath to induce FS [7], we have performed long-term prospective studies. Briefly, postnatal 10 days rats in the FS group (*n*=8) were placed into 44.9°C water for 5 min. Rats were taken out of the water immediately after seizure occurred, and the incidence of seizure with 5 min was 100%. The classification and temporal progression of epileptic seizure was diagnosed by trained observers according to the RACIN scale [23] with the following stages: (1) facial nerve tics only; (2) nodding spasm; (3) muscular spasm and clonus of forelimbs; (4) holotonia; and (5) generalized tonic-clonic seizure. At the end of observation period, all rats survived and they were gently toweled dry, placed beneath a warm condition at room temperature (21–22°C) until its fur appeared free of moisture, and returned to the cage. Water immersion was carried out once per day for consecutive 5 days. Rats without any treatment served as control (*n*=8). All animal experiments were performed in accordance with the guidelines approved by the Animal Studies Committee of Hangzhou Red Cross Hospital.

### Drug preparation and animal treatment

The compositions that listed in Table 1 were taken according to their proportion and Fangjing decoction is prepared by decocting them altogether, with a final concentration of 1 g/ml. Differential scanning calorimetry and powder X-ray diffraction were carried out on Fangjing Decoction to assess its stability [24], and it was demonstrated that no recrystallization or phase separation occurred during storage, manifesting a stable system. To explore the underlying functions of Fangjing Decoction in FS-induced brain injury, a total of 40 SD rats were randomly divided into 5 groups (*n*=8 per group) as follows: control group, FS group, FS group treated physiological saline (5 g/kg/d, FS + PS), FS group treated with Fangjing Decoction (5 g/kg/d, FS + FJD), and FS group treated with Fangjing Decoction (5 g/kg/d) and 300 nM Akt specific activator, SC79, (FS + FJD + SC79). Rats were administered with FJD (5 g/kg/d) according to their body weight by gavage for consecutive 7 days, and FS was induced from the 8th day with heated water-bath for another 5 days. The escape latency of seizure was measured as the interval between the moment the rats were placed in the water and the first sign of seizure onset within 5 days, while the duration of seizure was determined as the interval between seizure onset and termination. In addition, seizure frequency was also detected. Finally, the brains were removed for the following experiments.

### Apoptotic cell analysis by *in situ* terminal dUTP nick end-labeling

Hippocampal neuron apoptosis was identified by the terminal dUTP nick end-labeling (TUNEL) method using ApopTag *In Situ* Cell Apoptosis Detection Kit I (Wuhan Boster Co., Ltd., China) [25]. Rat hippocampal tissues were taken and embedded with paraffin and the hippocampal tissue slices were prepared. Then the sections were dewaxed, blocked in methanol containing 3% hydrogen peroxide, and treated with 0.1%Triton, followed by TUNEL staining (Roche Applied Science, Mannheim, Germany). The imaging was done with a fluorescence microscope (Nikon Instruments Inc., Brighton, MI, U.S.A.) to observe the results. Five views were randomly chosen under the microscope for each treatment group to count the number of TUNEL positive cells and total cells. The apoptotic rate was calculated by dividing the number of TUNEL positive cells by the total number of cells.

### Western blot analysis

Protein samples extracted from the hippocampus tissues were loaded to a 12% SDS–PAGE. Following electrophoresis, the proteins were transferred from the gel to PVDF membranes using an electric transfer system. The membranes were blotted with 5% skimmed milk in tris-buffered saline tween (TBST) for 2 h and then incubated with antibodies against Bax, Akt, phospho-Akt (p-Akt), mTOR, p-mTOR, and β-actin (1:1000; Cell Signaling Technology Boston, MA, U.S.A.) overnight at 4°C. After being washed three times with TBST, the PVDF membranes were incubated for 2 h at room temperature with HRP-conjugated secondary antibodies against IgG (1:5000; Peprotech, Rocky Hill, NJ, U.S.A.). Finally they were evaluated using the ECL Western Detection Reagents. The intensity of the tested protein bands was normalized to the internal reference.

### Measurement of GABA concentration

Hippocampus was dissected and separately homogenized. The centrifuged supernatants of each sample were used to measure the GABA levels by using the GABA ELISA Kit (Novatein Biosciences, Cambridge, MA; sensitivity 2.5–80 ng/mg) according to the manufacturer’s instructions while the protein concentration in each supernatant was determined with BCA protein assay (Thermo Fisher Scientific, Rockford, IL, U.S.A.).

### Statistical analysis

All analyses were performed using SPSS version 22.0 (SPSS, Chicago, IL, U.S.A.). Two-sided Student’s *t* test was used to compare differences between groups. All results were presented as mean ± standard deviation and *P-*value <0.05 was statistically considered significant.

## Results

### Fangjing decoction shortened escape latency and duration of FS and decreased the frequency in a FS rat model

**Figure 1:**
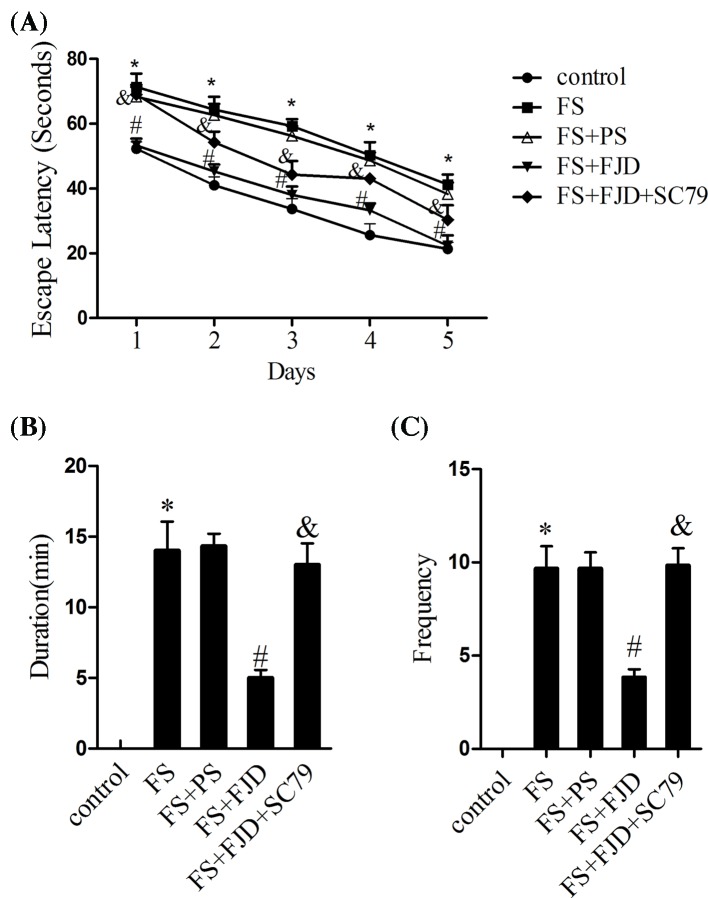
Effect of Fangjing decoction administration on the escape latency, duration and the frequency of FS in a FS rat model (**A**) The escape latency of recurrence of FS during 5 days in the control group, FS group, FS group treated with 5 g/kg physiological saline (FS + PS), FS group treated with 5 g/kg Fangjing decoction (FS + FJD), and FS group treated with 5 g/kg FJD and 300 nM Akt specific activator, SC79 (FS + FJD+ SC79). (**B**) The duration of FS during 5 days in the above subgroups. (**C**) The frequency of FS during 5 days in the above subgroups. *n*=8 per group. ^*^P<0.05 vs the control group; ^#^*P*<0.05 vs the FS + PS group; ^&^*P*<0.05 vs the FS + FJD group.

To study the effect of Fangjing decoction on the degree of convulsion in rats with FS, we first contrasted the escape latency, duration, and frequency of seizures. The time to seizure onset, also described as the escape latency (Figure 1A; *P*<0.05), the duration (Figure 1B; *P*<0.05), and frequency of FS (Figure 1C; *P*<0.05) in FS rat model, increased significantly compared with the control group, but significantly reduced in FS + FJD group as compared with the FS + PS group (Figure 1, *P*<0.05). However, the SC79 treatment in FS + FJD group promoted the recurrence of FS in rats since the escape latency, duration, and frequency of FS were observably increased (Figure 1, *P*<0.05). Taken together, Fangjing decoction could effectively shorten the escape latency and duration of recurrence of FS, decline the frequency of outbreaks, and lessen the degree of FS.

### Fangjing decoction suppressed hippocampal neuron apoptosis in FS rats

**Figure 2:**
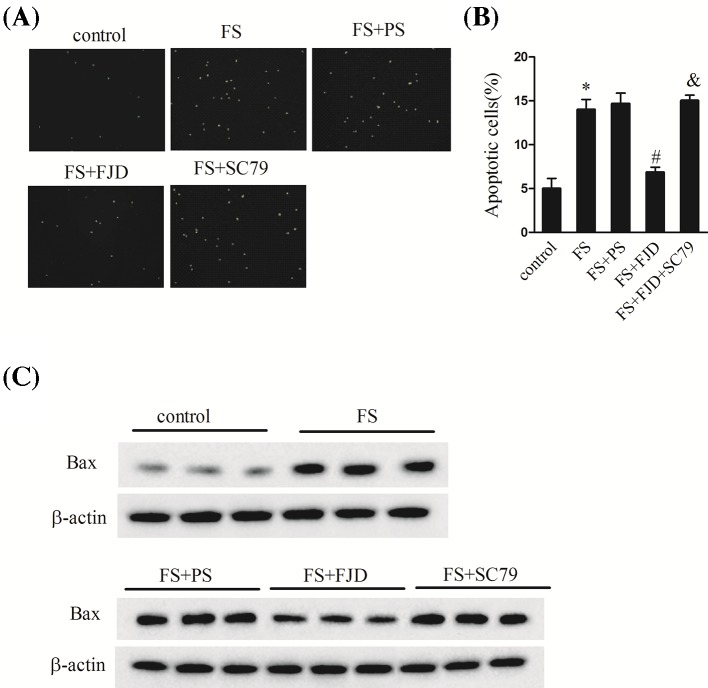
Effect of Fangjing decoction administration on hippocampine neuron apoptosis in a FS rat model (**A**) The picture of TUNEL assay. (**B**) The percent of apoptotic hippocampus neural cells in the groups of control, FS, FS + PS, FS + FJD, and FS + FJD + SC79. (**C**) The protein levels of Bax in the groups of control, FS, FS + PS, FS + FJD, and FS + FJD + SC79. *n*=8 per group. ^*^P<0.05 vs the control group; ^#^*P*<0.05 vs the FS + PS group; ^&^*P*<0.05 vs the FS + FJD group.

We further explored the biological functions of Fangjing decoction in hippocampal neuron apoptosis. As shown in Figure 2, few TUNEL-positive apoptotic neurons were found in the hippocampus of control group (Figure 2A,B). In the FS group, the percentage of apoptotic neurons (Figure 2B, *P*<0.05) and apoptosis protein Bax levels (Figure 2C, *P*<0.05) in the hippocampus were memorably increased compared with the control group. In comparison with the FS + PS group, the percentage of apoptotic neurons (Figure 2B, *P*<0.05) and Bax protein levels (Figure 2C, *P*<0.05) in the hippocampus were clearly reduced in the FS + FJD group, while neuronal apoptosis (Figure 2B, *P*<0.05) and Bax levels (Figure 2C, *P*<0.05) were significantly increased after SC79 treatment when compared with the FS + FJD group (*P*<0.05). These findings showed that Fangjing decoction administration could alleviate neuronal apoptosis in the hippocampus of FS rats, while SC79 administration exacerbated neuronal apoptosis in the hippocampus following FS.

### Fangjing decoction inhibited the activation of Akt/mTOR signaling in FS rat hippocampus

**Figure 3:**
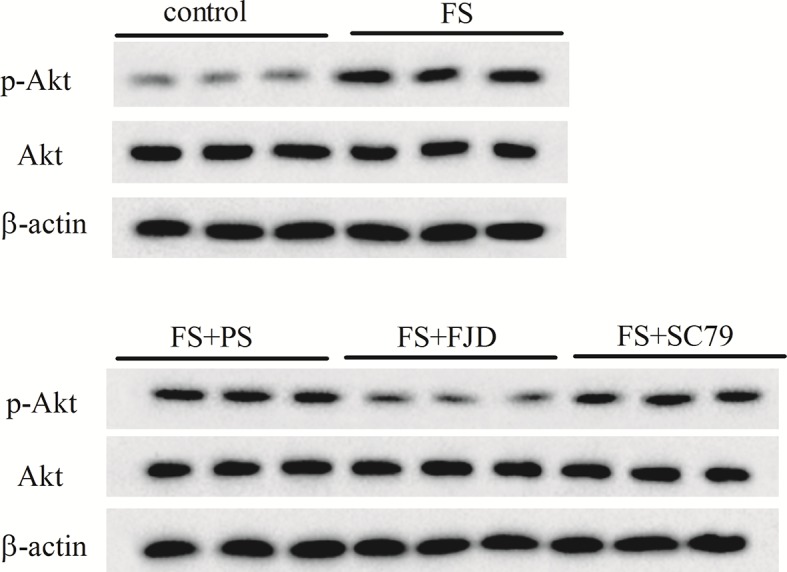
The protein levels of hippocampus p-Akt and Akt in the groups of control, FS, FS + PS, FS + FJD, and FS + FJD +SC79 *n*=8 per group.

**Figure 4:**
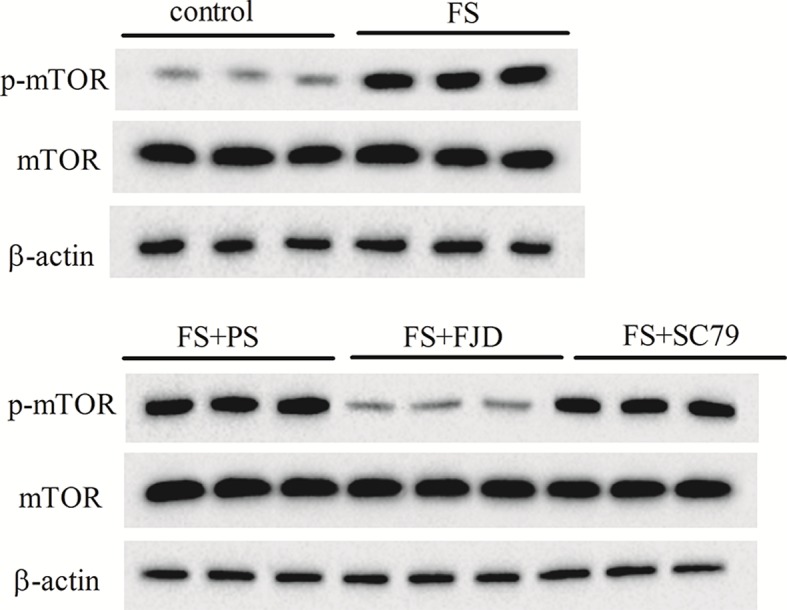
The protein levels of hippocampus p-mTOR and mTOR in the groups of control, FS, FS + PS, FS + FJD, and FS + FJD+ SC79 *n*=8 per group.

To further understand the molecular mechanism by which Fangjing decoction inhibited hippocampine neuron apoptosis, we measured the p-Akt and p-mTOR protein levels in rats following FS and with Fangjing decoction administration, since Akt/mTOR signaling pathway activation played an important role in epileptogenesis. Western blot analysis showed that the protein levels of p-Akt (Figure 3) and p-mTOR (Figure 4) increased in rat hippocampus following FS. Compared with the FS + PS group, Fangjing decoction administration markedly down-regulated the levels of p-Akt (Figure 3) and p-mTOR (Figure 4) in FS rat hippocampus, while the expression levels of p-Akt (Figure 3) and p-mTOR (Figure 4) were significantly up-regulated in the FS + FJD + SC79 group. These results demonstrated that Fangjing decoction reduced the activation of Akt/mTOR signaling that had been activated in rat hippocampus by FS.

### Fangjing decoction elevated GABA content in FS rat hippocampus

**Figure 5:**
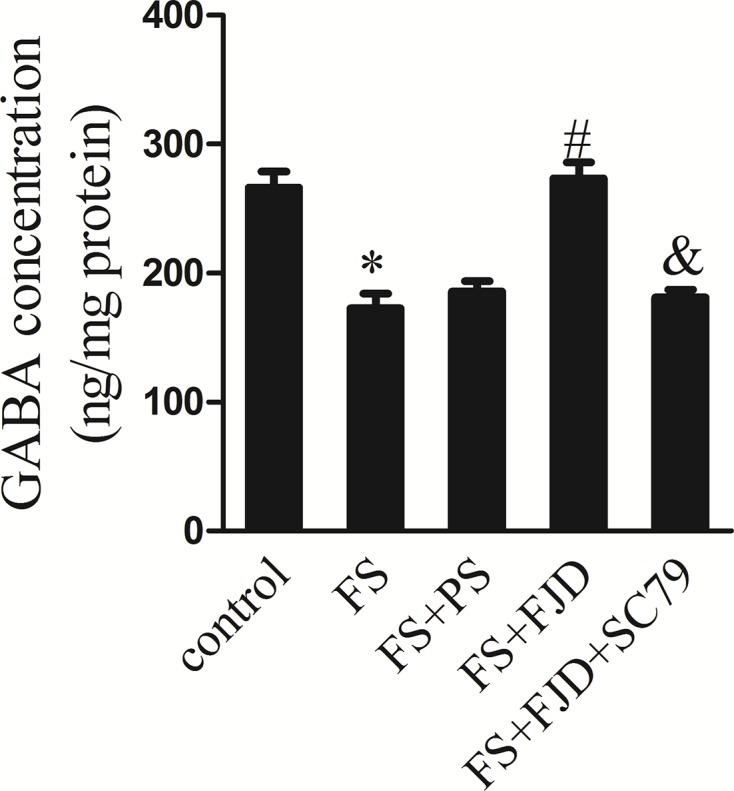
The hippocampus GABA concentration in the groups of control, FS, FS + PS, FS + FJD and FS + FJD + SC79 *n*=8 per group. ^*^P<0.05 vs the control group; ^#^*P*<0.05 vs the FS + PS group; ^&^*P*<0.05 vs the FS + FJD group.

GABA was the most abundant inhibitory neurotransmitter in the mammalian central nervous system and low expression of GABA was responsible for seizure onset. We thus explored whether Fangjing decoction could affect GABA expression. As a result, the content of GABA in the FS group were remarkably lower than that in the control group, while Fangjing decoction treatment resulted in an increase in GABA content in comparison with the FS + PS group (Figure 5, all *P*<0.01). In contrast, SC79 administration led to an opposite effect (Figure 5, all *P*<0.01). Collectively, Fangjing decoction treatment increased GABA levels in hippocampus of FS rats.

## Discussion

In the present study, our findings revealed that Fangjing decoction administration significantly reduced the escape latency, the duration, and the frequency of FS in an FS rat model. Additionally, Fangjing decoction treatment inhibited hippocampus neuron apoptosis via suppressing the activation of Akt/mTOR signaling pathway as well up-regulating GABA concentration in rats following FS. These results provide further evidence indicating that Fangjing decoction has anticonvulsant properties.

It is well known that understanding neuronal apoptotic mechanisms is of great significance in developing novel therapies for neurodegenerative diseases or other central nervous system-related diseases [26,27]. Specifically, neuronal apoptosis resulting from FS occurs partially from excessive Na^+^ and Ca2^+^ entry. Previous studies have demonstrated that the release of intrinsic cytochrome complex led to activation of the death receptors, TNF/caspase-8 pathway and Bax signaling, thereby resulting in neuronal cell death [28]. Therefore, antineuronal apoptotic therapy has been an attractive strategy to combat FS-induced cerebral lesion. Fangjing decoction, a classical Traditional Chinese Medicine prescription, has been demonstrated to be effective for the treatment of experimental FS [13]. However, little is known about the mechanism of its neuroprotective action.

In the present paper, we utilized the rat FS model to explore the neuroprotective mechanism of Fangjing decoction, and found that Fangjing decoction administration could shorten the escape latency and the duration of recurrence of FS, decline the frequency of outbreaks, and lessen the degree of FS. This finding is consistent with our previous study. These data are in accordance with our previous studies showing that Fangjing decoction could prolong the latency of recurrence of febrile convulsions, shorten duration, and lessen the degree of convulsions [13]. The present study further found that Fangjing decoction treatment largely decreased the percentage of apoptotic neurons and Bax protein expression, reversing the apoptotic status induced by FS. Proapoptotic mediators produced in hippocampus neural cells, such as Bax, caspase-3, TLR4, NF-κB and Akt as well as m-TOR, play an important role in neuro-apoptosis, thus, the release of these cytokines is considered as an indicator for neurological damage [29]. Especially, the Akt/mTOR signaling pathway can be activated when cells are stimulated by growth factors to control the growth, proliferation, survival, and apoptosis of various cells; therefore, Akt/mTOR signaling pathway plays an important role in promoting the progression of several diseases [30]. The increases in hippocampal levels of p-Akt and p-mTOR in FS rats were significantly attenuated following Fangjing decoction treatment for 5 days. Taken together, these data suggest that Fangjing decoction can ameliorate FS-induced cerebral injury through inhibiting neuronal apoptosis and controlling the Akt/m-TOR signaling pathway. As with other forms of epilepsy, dysfunctions in the brain GABA system are thought to have a crucial role in the pathogenesis of seizures [31]. In particular, many studies in human cohorts affected by seizures have highlighted potential abnormalities related to various genes coding for proteins of the GABAergic system, including transporters, synthetizing enzymes, and subunits of both GABA A and GABA B receptor subtypes [32]. In the present study, we also found that Fangjing decoction treatment resulted in an increase in GABA concentration in hippocampus of FS rats, suggesting that Fangjing decoction might up-regulate GABA levels to relieve FS-induced brain injury.

In conclusion, the results of the present study show that the Chinese herbal prescription Fangjing decoction had anticonvulsant effects. We also provide evidence that Fangjing decoction exerts its observed antiseizure effects by inhibiting neuronal apoptosis and activation of Akt/mTOR pathway, and simultaneously increasing hippocampus GABA contents. These findings may provide strong evidence for developing more effective anticonvulsant drugs.

## Abbreviations

Akt: protein kinase B
FS: febrile seizures
GABA: γ-aminobutyric acid
HRP: horseradish peroxidase
IgG: immunoglobulin G
mTOR: mammalian target of rapamycin
NF-κB: nuclear factor kappa B
p-Akt: phospho protein kinase B
p-mTOR: phospho-mTOR
SD: Sprague–Dawley
SPF: specific pathogen free
TBST: tris-buffered saline tween
TNF: tumour necrosis factor
TUNEL: terminal dUTP nick end-labeling
