# Correction: Fangjing decoction relieves febrile seizure-induced hippocampal neuron apoptosis in rats via regulating the Akt/mTOR pathway

**DOI:** 10.1042/BSR-20181206_COR

**Published:** 2019-01-22

Bioscience Reports (2018) 38(5), BSR20181206; https://doi.org/10.1042/BSR20181206

The published article contains several errors listed as follows:

1. In the Abstract, “Compared with the control group (*n*=8), Fangjing decoction effectively shortened escape latency and duration of FS and decreased the frequency of FS in rats (*n*=8)” should read “Compared with the control group (*n*=8), Fangjing decoction effectively prolonged the latency but shortened the duration of FS in rats (*n*=8)”.
2. In the ‘Methods’ section under Drug preparation and animal treatment, “The escape latency of seizure” should read “The latency of seizure”. The penultimate sentence “In addition, seizure frequency was also detected” should be removed.
3. Under ‘Results’ section, the subsection “Fangjing decoction shortened escape latency and duration of FS and decreased the frequency in a FS rat model” should be replaced with the following:

## Fangjing decoction prolonged the latency but shortened the duration of seizures in a FS rat model

To study the effect of Fangjing decoction on the degree of convulsion in rats with FS, we firstly contrasted the latency and duration of seizures. The time to seizure onset, also described as the latency, was increased significantly in FS rat compared with the control group, but it was further prolonged in FS + FJD group (Figure 1A; *P*<0.05). The duration of seizure was long in FS rat model, but it was shortened by Fangjing decoction in FS + FJD group (Figure 1B; *P*<0.05). However, the SC79 treatment in FS + FJD group promoted the recurrence of FS in rats since the latency was reduced and duration of seizure was notably prolonged (Figure 1A and B, *P*<0.05). Taken together, Fangjing decoction could effectively prolong the latency and shorten the duration of FS, indicating its efficacy in alleviating seizure.

**Figure 1:**
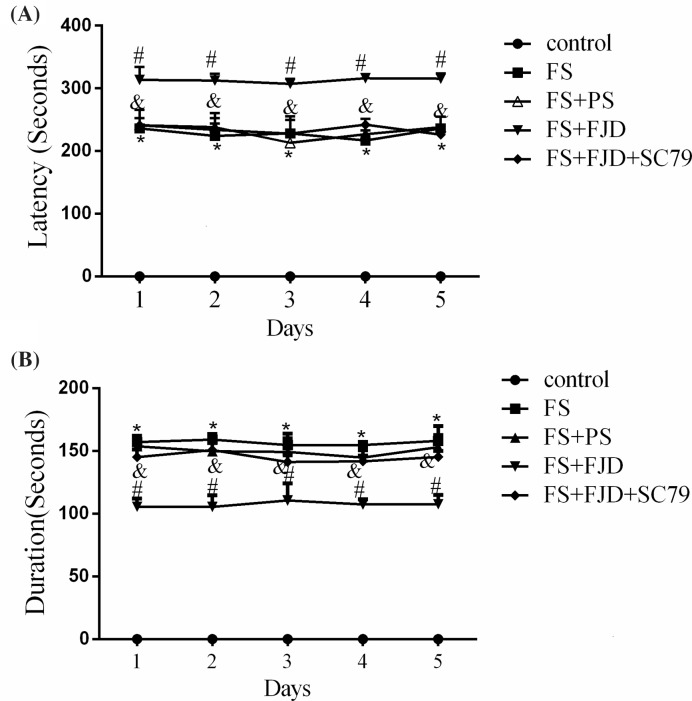
Effect of Fangjing decoction administration on the latency and duration of FS in a FS rat model (**A**) The latency of FS during 5 days in the control group, FS group, FS group treated with 5 g/kg physiological saline (FS + PS), FS group treated with 5 g/kg Fangjing decoction (FS + FJD) and FS group treated with 5 g/kg FJD and 300 nM Akt specific activator, SC79 (FS + FJD + SC79). (**B**) The duration of FS during 5 days in the above subgroups; *n*=8 in each group. *P<0.05 vs. the control group; ^#^*P*<0.05 vs. the FS + PS group; ^&^*P*<0.05 vs. the FS + FJD group.

4. The corrected Figure 1 and its legend are presented in this correction.
5. In the ‘Discussion’ section, “In the present study, our findings revealed that Fangjing Decoction administration significantly reduced the escape latency, the duration and the frequency of FS in an FS rat model” should read “In the present study, our findings revealed that Fangjing decoction administration significantly prolonged the latency and shortened the duration of FS in a FS rat model”.
  Furthermore, “In this paper, we utilized rat FS model to explore the neuroprotective mechanism of Fangjing Decoction, and found that Fangjing Decoction administration could shorten the escape latency and the duration of recurrence of FS, decline the frequency of outbreaks and lessen the degree of FS” should read “In this paper, we utilized rat FS models to explore the neuroprotective mechanism of Fangjing decoction, and found that Fangjing decoction administration could prolong the latency and shorten the duration of FS”.

